# Reasons and multilevel factors associated with unscheduled contraceptive use discontinuation in Ethiopia: evidence from Ethiopian demographic and health survey 2016

**DOI:** 10.1186/s12889-019-8088-z

**Published:** 2019-12-27

**Authors:** Kibrom Taame Weldemariam, Kebede Embaye Gezae, Haftom Temesgen Abebe

**Affiliations:** 1 Department of Biostatistics, School of Public Health, College of Health Sciences, Aksum University, P.O.Box: 298, Axum, Ethiopia; 2 Department of Biostatistics, School of Public Health, College of Health Sciences, Mekelle University, Mekelle, Ethiopia

**Keywords:** Contraceptive, Discontinuation, Ethiopia, Factors, Health, Multilevel, Survey

## Abstract

**Background:**

Contraceptive discontinuations for reasons other than the desire for pregnancy are a public health concern because of their negative effect on reproductive health outcomes. In Ethiopia, the contraceptive discontinuation rate is increasing; however the factors associated are poorly understood. So this study was aimed at assessing reasons and multilevel factors for unscheduled contraceptive use discontinuation.

**Methods:**

This is a cross-sectional study of Ethiopian women who participated in the Ethiopian demographic health survey from January 18, 2016, to June 27, 2016. Ever using any contraceptive with in the calendar of the survey were an inclusion criteria for which 3835 women were found eligible. The data were analyzed using multilevel binary logistic regression in STATA version 14. Variables with *p*-value less than 0.05 were considered as statistically significant, and reported using adjusted odds ratio and 95% confidence interval. Median odds ratio and interval odds ratio, to quantify the magnitude of the general and specific contextual effect respectively, were used. Receiver operating characteristics curve and akaike’s information criterion were used for model comparison.

**Result:**

The prevalence of unscheduled contraceptive use discontinuation was 46.18% for the principal reason of method related problems (Side effects-45.3%, needing better method-33.6%, and inconvenience-21.1%,). Women heading a household (AOR = 1.281, 95%CI 1.079–1.520), women who had no work (AOR = 0.812, 95%CI 0.673, 0.979) compared to professionals, living in poorest house hold income (AOR = 0.753, 95%CI 0.567, 0.997) compared to middle, residing in community with low contraceptive utilization rate (AOR = 1.945, 95%CI 1.618, 2.339), residing in poor community (AOR = 0.763, 95%CI 0.596–0.997), and having more children, and region were found to be significant predictors of unscheduled contraceptive use discontinuation.

**Conclusion:**

Method related problems were found to contribute for more than half of the contraceptive use discontinuation. Both individual and community level factors were found to significantly influence the Unscheduled contraceptive use discontinuation. The outcome was common in groups who could have more social interactions and knowledge on which myths and rumors are common. So strengthening the efforts to reduce contraceptive use discontinuation and quality of contraceptive service provision could be important.

## Introduction

Discontinuation of contraception method is an abandonment of contraceptive method utilization among women who ever use contraception for any reason [1]. Women with unmet need are defined as those who want to stop or delay childbearing but are not using modern contraceptive methods [2]. Unscheduled contraceptive use discontinuation is an abandonment of contraceptive utilization despite the desire to avoid pregnancy.

Although there is an increase in contraceptive prevalence rate (CPR), worldwide 38% women using family planning discontinue their contraceptive method without switching to another method, despite their desire to avoid pregnancy [3]. 64% women discontinued their contraceptive use by the 36th month [4]. Greater than a half of women who start using a modern contraceptive method stop using it before two years of continual utilization [1].

Discontinuation for reasons other than wanting to become pregnant has a big contribution to unplanned pregnancies, unwanted births, unwanted fertility and termination of pregnancies that may be done through unsafe abortion [2, 5].

Tackling discontinuation has been highlighted as a key global health issue [6]. However; a main obstacle to reduce unwanted fertility is the discontinuation of modern contraception [1]. One-third of unintended pregnancies are due to method failure or discontinuation [7, 8]. In countries with moderate to high contraceptive prevalence, the majority of unintended pregnancies are the result of contraceptive discontinuation or failure [9].

Investing in family planning accelerates achievement across the five Sustainable Development Goal (SDG) themes which are the five P’s (People, Planet, Prosperity, Peace, and Partnership) [2]. So if FP2020 is to reach an additional 120 million women with an unmet need for family planning by 2020, family planning programs need to escape their selves from becoming a “leaking bucket” [10] in order to effectively addressed contraceptive discontinuation.

Therefore; promotion of continuation rates and re adoption among past users is better than promotion of new acceptance rates, because unwanted and mistimed pregnancies would increasingly result from discontinuation of methods rather than not ever using contraceptive at all [11]. Contraceptive discontinuation for reasons other than the desire for pregnancy is a public health concern due to its negative reproductive health outcomes consequences [12].

In sub-Saharan Africa countries, even though contraceptive use is rising, contraceptive discontinuation rates are also highly increasing [13]. According to the World Bank collection of development indicators, compiled from officially recognized sources, contraceptive prevalence among women ages 15–49 in Ethiopia was reported at 35.9% in 2016. So this indicates a moderate contraceptive prevalence rate during the year of 2016.

In the study done in 2015, 25% of reproductive age women had discontinued their contraceptive utilization [14], whereas the study done in 2017 stated that 27.4% of women discontinued their contraceptive utilization within 24 months [15]. In EDHS 2016, the overall discontinuation rate, including for the reason of wanting to become pregnant, was 35%. So, discontinuation rate of modern contraceptive, in Ethiopia, is increasing. Despite a high level of knowledge majority of male and female showed negative attitude toward the contraceptive practicing [16].

In Ethiopia, almost all studies done on contraceptive use discontinuation were based on a single level analysis, however; the multilevel factors that could affect the contraceptive use discontinuation, using multilevel analysis, are not well addressed. For data with a hierarchical nature, unlike single level analysis, using multilevel models that allow one to account for the clustering of subjects within clusters of higher-level units when estimating the effect of subject and cluster characteristics on subject outcomes, would give us appropriate parameter estimation [17]. Limited evidences are available regarding multilevel predictors of contraceptive use discontinuation in Ethiopia at nationally representative sample.

Moreover; the studies that have been done on factors associated with contraceptive discontinuation was done on all women including those discontinued for the reason of wanting to get pregnant and on all episodes of discontinuation; so this could lead to complex results. For example, one could conclude that the odds of discontinuation is higher in the age group of 25–34 than of 15–24, but this might be due to higher need of to be pregnant in the 25–34 age group. Using data from overlapping contraceptive calendars, more than one-third of women were discordant in their reports for the reference month in the two surveys; and women using with more complex reproductive histories, including more births and more episodes of contraceptive use, were least likely to report reliably [18]. Therefore; this study uses the last method discontinued in the last five years prior to the survey to increase reliability by reducing recall bias. There were also additional factors like reasons for contraceptive discontinuation collected in EDHS-2016.

So this study has taken a step further from the routine EDHS report by further analysis of DHS data using advanced analysis model to assess reasons and multilevel factors of unscheduled contraceptive use discontinuation using a multilevel logistic regression model and provides context specific information to program planners and policy makers.

**Fig. 1:**
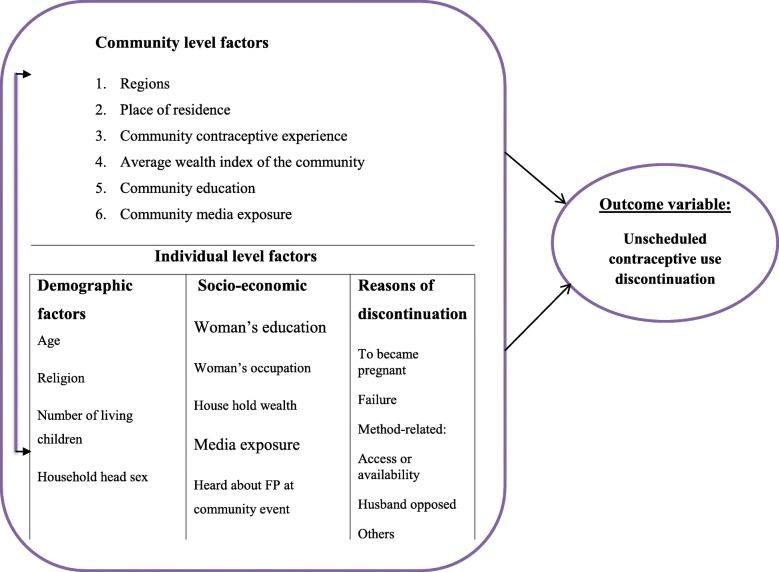
Conceptual frame work of unscheduled contraceptive use discontinuation that conceptualizes the relationship of individual and community level predictors to the outcome variable in Ethiopia, January 2018. Source: adapted from different literatures

For conceptual framework (refer Fig. 1).

## Methods and materials

### Study area

The study was conduct in Ethiopia in 2018 from EDHS 2016. According to Ethiopia Demographics Profile 2018, Ethiopia is the second-most populous nation on the African continent with 105,350,020 estimated populations and with 79.6% of its population living in rural areas. Administratively, Ethiopia is divided into nine geographical regions and two administrative cities. Ethiopia has been doing more efforts to increase family planning utilization and continuation through increasing the number of skilled providers delivering high-quality contraceptive services and to ensure access for all populations although there is no discontinuation specific effort [19].

### Study design

A cross sectional study design was used to collect data from the EDHS 2016 data set for the current analysis.

### Population

#### Source population

Reproductive age women who ever used contraceptive in the 5- years prior to the EDHS 2016.

#### Study population

Reproductive age women in the selected clusters who ever used contraceptive in the 5- years prior to EDHS 2016.

### Eligibility criteria

#### Inclusion criteria

All reproductive age women who ever used contraceptive in the 5-years prior to EDHS 2016.

#### Exclusion criteria

Women of reproductive age who discontinued contraceptive use for the reason of wanted to become pregnant.

### Sample size determination, and sampling procedure

**Fig. 2:**
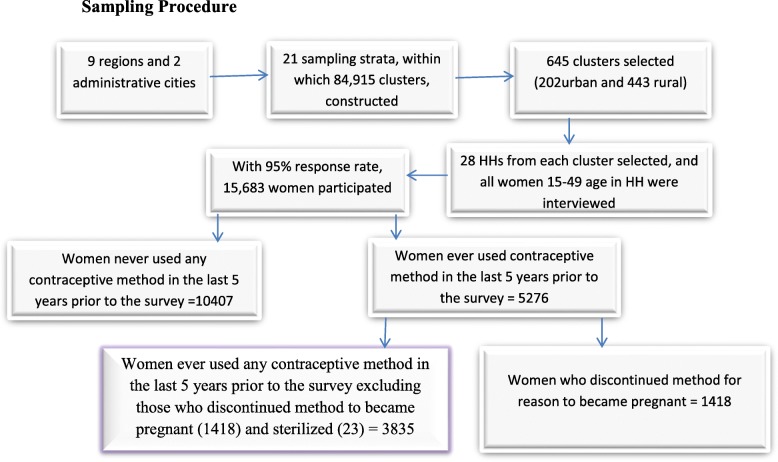
Schematic representation of sampling procedure for multilevel factors associated with unscheduled contraceptive use discontinuation in Ethiopia, January 2018. Source: CSA/Ethiopia, EDHS 2016

EDHS 2016 used a multistage stratified cluster sampling method. The sampling frame used was adopted from the Ethiopia Population and Housing Census of 2007 that had complete list of 84,915 enumeration areas. 2016 EDHS sample was selected in two stages. Each region was stratified into urban and rural areas, yielding 21 sampling strata. Samples of enumeration areas were selected independently in each stratum in two stages. At second stage 28 households per cluster were selected. All women of 15–49 ages, in the selected households, were interviewed. A total of 15,683 women were successfully interviewed, yielding a response rate of 95% [20]. Schematic representation of sampling procedure (refer to Fig. 2).

### Study variable

#### Dependent variable

The dependent variable is unscheduled contraceptive use discontinuation in the 5 years prior to the survey of 2016. This dependent variable was coded to have a binary response to the question “last method discontinued in the last 5 years” if discontinued “yes = 1” and “otherwise = 0”. This outcome variable was then modeled to historical predictor variables that were selected based on existing evidences.

#### Independent variables

##### Individual level variables

Respondent’s age group, respondent’s occupation, respondent’s education, Religion, Number of living children, heard about family planning at community -event/conversation, Household head sex, House hold wealth, and media exposure on family planning messaging.

##### Community level variables

In addition to region, and place of residence, the aggregated values that are assumed to affect group level variation which are: Community contraceptive, average wealth index of the community, community education, and community media exposure on family-planning messaging were included.

The aggregated community level predictor variables were constructed by aggregating individual level values at cluster level, and binary categorization of the aggregated variables were done based on the distribution of the proportion values calculated for each cluster (community). Mean for normally distributed or median for not normally distributed community level aggregated predictor variables were used as cut off point for categorization. Histogram was used to check the distribution whether it is normal or not.

Community media exposure was categorized as exposed if the proportion of women in the community exposed to media was > = 26.087% and categorized as not exposed if the proportion was 0–26.087%. Community education was classified as literate if the proportion women in a community attending high-school and above was > = 12.5% and as illiterate if the proportion was 0–12.5%. Community wealth was categorized as rich if the proportion of women with richer and richest wealth quintile in one community was > = 43.478% and as poor if the proportion was 0–43.478%. Community contraceptive rate was classified as high if the proportion of women utilizing contraceptive in a community was > = 20.833% and as low if the proportion was 0–20.833%.

### Operational definition

Unscheduled discontinuation: Unscheduled discontinuation of contraception method is an abandonment of contraceptive method utilization without switching to another method, despite their desire to avoid pregnancy.

From the reasons listed as a cause of discontinuation in EDHS-2016: discontinuation was classified in to two: Scheduled discontinuation: women who discontinued due to the need to become pregnant and Unscheduled discontinuation: women who discontinued for the reason other than desire to become pregnant. This unscheduled discontinuation were examined in six sub-categories: Failure (became pregnant while using), Method-related: (side effects, wanted a more effective method and inconvenient to us), Access (availability or cost), Unreliable reasons (Infrequent sex/husband away, Marital dissolution/separation, difficult to get pregnant/ menopausal): those are classified under unscheduled discontinuation because they are among the causes for failure and unwanted pregnancy and they are still didn’t want to become pregnant, other reasons: (other, fatalistic, don’t know) and husband opposed.

Cluster or community: means an enumeration area.

Reproductive age women: are women who are from 15 to 49 years old.

### Data collection, management and quality control

The survey tool had 5 parts that are household questionnaire, woman’s questionnaire, man’s questionnaire, biomarker questionnaire and health facility questionnaire. The EDHS survey questionnaire assesses the household and respondent characteristics, fertility and family planning, maternal and child health, nutrition, HIV/AIDS, sexual violence, and others.

After the permission to download dataset of EDHS-2016 has been received through electronic mail, selecting and extracting important variables related to contraceptive use discontinuation were performed from the data set.

Further data cleaning, labeling, coding and recoding were done for all selected variables. In addition categorization was done for continuous and categorical variables using information from different literatures or based on their clinical and public health importance.

### Analysis

The data was taken from EDHS 2016, and analyzed using multilevel logistic regression in STATA version 14. In this study which assessed the effect of different predictors on the unscheduled contraceptive use discontinuation, only women of reproductive age (15 to 49 years old) who ever use contraceptive or discontinue a contraceptive use (recent one) for reasons other than desire to become pregnant, which is a public health interest, were included. Because the difference in discontinuation among different categories may be due to the difference of desire to become pregnant across those categories. Thus; women who discontinued for the reason of need to be pregnant as well as sterilized women, who didn’t have history of discontinuation before sterilization, were excluded from the analysis. Why we prefer the recent discontinuation is for easily remembrance of the discontinued method and its reason (to reduce recall bias).

#### Descriptive statistics

Frequency and percentage were used to report categorical variables; mean for continuous normal variables, and median followed by Inter quartile Range, for continuous explanatory variables that violate assumption of normality, were also used. In addition cross tabulation was done to show the proportion of different categories of each characteristic with respect to the outcome variable (contraceptive use discontinuation).

#### Multilevel logistic regression analysis

Multilevel analysis can address the two main problems that can occur either due to aggregation or disaggregation of data. If data are aggregated we loss data and power, on the other hand if data are disaggregated and are not independent of one another, it can lead to false positive significant effect when in fact not exist [21].

Therefore; this study has an individual level dependent variable with dichotomous nature and individual and community level independent variables. Thus; we prefer multilevel logit regression analysis, for the last method discontinued, that simultaneously investigates community and individual level factors and reflects their effect on individual level outcome and therefore; the model produces appropriate estimates about the parameters (refer Table 1).

**Table 1:** Types of studies based on the nature of variables ( [21])

Independent Variable	Dependent Variable	Type of Study
Group-Level	Group-Level	Ecological
Individual-Level	Individual-Level	Individual-Level
Group & Individual Level	Individual-Level	Multilevel

#### Bivariate multilevel logistic analysis

Bivariate regression analysis was performed to explore association between dependent variable and a wide range of independent variables to identify the potential variable with *p*-value of 0.25 and less to be entered to the final model [22].

#### Multivariable multilevel logistic analysis

Variables with p-value of less than 0.05 in the final model were considered as significant predictors of contraceptive use discontinuation. Adjusted odds ratio (AOR) and 95% confidence interval (CI) were used to report the findings of this study. Variance partition coefficient (VPC), proportional change in variance (PCV) and the median odds ratio which are measures of components of variance and heterogeneity in outcome were used to quantify the magnitude of the general contextual effect. To integrate the higher level fixed effect and the random residual variations, 80% interval odds ratio (IOR-80) which is summary measures of effect for cluster-level covariates was also used [23].

#### Model fit statistics

Receiver operating characteristics (ROC) curve was used to assess general accuracy of the model to the data set using the area under receiver operating characteristics (AUC). Relative goodness-of-fit test was conducted using akaike’s information criteria (AIC) for each of the models and compared [24]. AIC is better in situations when a false negative finding would be considered more misleading than a false positive (for sensitive model), and BIC is better in situations where a false positive is as misleading as, or more misleading than, a false negative (for specific model) [25].

#### Model diagnostics

Multi-collinearity diagnostic evaluation was done using variance inflation factor (VIF), and thus value of VIF greater than 10, gives evidence of multi-collinearity [26]. The level-2 random residual was tested for its normal distribution.

#### Model specification

The focus of this study was on the random effects intercept, because I theorized only one individual level variable to vary its effect on the outcome across clusters (which is women education-but this random slope fitted was found to be insignificant, so not included in the final model, see Additional file 1).

**Level 1**: Model for individual level predictors (fixed effects):

$$ Log\ (odds)={\beta}_{0j}+{\beta}_{1j}{x}_{1 ij}+{\beta}_{2j}{x}_{2 ij}+...+{\beta}_{Kj}{x}_{Kij}+{e}_{ij}. $$

**Level 2**: Model for intercept and slopes (random effects):

*Mixed model* : *Log*(*odds*) = *β*_00_ + (*B*_10_ + *u*_1*j*_) ∗ *x*_*ij*_ + *β*_01_ ∗ *X*_*j*_ + *u*_0*j*_ + *e*_*ij*_.

$$ Log(odds)={B}_{00}+\left({\beta}_{10}+{u}_{1j}\right)\ast {x}_{ij}+{\beta}_{01}\ast {X}_j+{\beta}_{11}\ast {x}_{ij}\ast {X}_j+{u}_{0j}\left( if\ interaction\ is\ assumed\right). $$

Where Log (*odds*) is the log-odds that the outcome variable equals one instead of zero (i.e. the chance that a pupil *i* from a cluster *j* discontinues contraceptive use), β_**00**_ is the fixed intercept, *u*_**0j**_ is the deviation of the cluster-specific intercept from the fixed intercept (i.e. the level-2 residual), **x**_**ij**_ refers to the level-1 variable, **X**_j_ refers to the level-2 variable, β _**10**_ … β _**K0**_ are the fixed slopes of **x**_**ij**_ (the level-1 variable), *u*_**1j**_ … **u**_**Kj**_ are the deviation of the cluster-specific slopes from the fixed slopes (i.e. the residual term associated with the level-1 variable) and β _**01**_ is the fixed slope of **X**_j_ (the overall effect of cluster level variable), β _**11**_ is the coefficient estimate associated with the cross-level interaction [27].

## Result

In this study, all the descriptive part tables and proportions are weighted unless indicated as un-weighted; because the EDHS data was coming from strata and clusters that are sampled disproportionately and for this reason the DHS manual recommends weighting to make the sample more representative.

### General description of the study participants

In this study, a total of 3835 women who ever tried any contraceptive in the 5 years prior to the survey, excluding women having scheduled contraceptive discontinuation, were included in the analysis. The participants were nested in 529 communities. The numbers of women nested per community were ranged from 1 to 19 with a median of 7 and inter quartile range of 5.

The mean age of the participants was 30.4 years (SD = 7.831), having a mean of 3.12 children (SD = 1.798) per woman. More than half (53.79%) of the participants were orthodox followers, more than one third (42.99%) had no any work, 76.33% were from rural residents, 54.22% were uneducated and 28.08% were from the richest group of household wealth index. The prevalence of unscheduled contraceptive discontinuation was 46.18%.

From the respondent’s occupation, discontinuation rate was higher (51.5%) among professional women. From the age category, older women were found to have highest contraceptive use discontinuation, whereas the younger women had lowest discontinuation rate. More than half (52.1%) of women heading a household and 47.7% of women exposed to media had contraceptive use discontinuation (see Table 2).

**Table 2:** Description and bivariate logistic analysis of individual level factors of unscheduled contraceptive discontinuation, EDHS 2016, January 2018

Individual Level Variables	Unscheduled Contraceptive Discontinuation			
	Yes (%)	No (%)	Total (%)	p-value
Age category				
15–24	404.1 (42.8)	539 (57.2)	943.1 (24.6)	0.001
25–34	785.8 (46.8)	894.2 (53.2)	1680 (43.8)	------
35–49	581.1 (47.9)	630.8 (52.1)	1211.9 (31.6)	0.609
Number of children				
< =1	494(42.6)	665(57.4)	1159(30.2)	< 0.001
> =2	1277(47.7)	1399(52.3)	2676(69.8)	------
Household head				
Male	1407.7 (44.9)	1729.6 (55.1)	3137.3 (81.8)	------
Female	363.4 (52.1)	334.3 (47.9)	697.7 (18.2)	0.001
Respondent’s occupation				
No occupation	716.3 (43.4)	932.5 (56.6)	1648.8 (43)	0.002
Professionals	550.5 (51.5)	519 (48.5)	1069.5 (28)	------
Services	78.2 (50.6)	76.4 (49.4)	154.6 (4)	0.688
Skilled manual	366.1 (44.2)	462.6 (55.8)	828.7 (21.6)	0.085
Unskilled manual	27.1 (51.4)	25.7 (48.6)	52.7 (1.3)	0.439
Others	33 (40.9)	47.7 (59.1)	80.7 (2.1)	0.082
Respondent’s education				
No education	950.1 (45.7)	1129 (54.3)	2079.1 (54.2)	0.537
Primary	545 (46.6)	623.3 (53.4)	1168.3 (30.5)	------
Secondary	148.3 (45)	181.1 (55)	329.4 (8.6)	0.762
Higher	127.6 (49.4)	130.5 (50.6)	258.2 (6.7)	0.708
Household wealth index				
Poorest	182.2 (41.6)	255.5 (58.4)	437.6 (11.4)	4.202
Poorer	320.6 (45.8)	379.1 (54.2)	699.7 (18.2)	2.210
Middle	376.4 (47.1)	423.1 (52.9)	799.5 (20.9)	------
Richer	370.8 (45.2)	450.4 (54.8)	821.2 (21.4)	1.147
Richest	521.1 (48.4)	555.9 (51.6)	1077 (28.1)	0.134
RELIGION				
Orthodox	1040 (50.4)	1023 (49.6)	2063 (53.7)	------
Catholic	7.9 (25.6)	23 (74.4)	30.9 (0.8)	0.508
Protestant	283.9 (31.8)	609.2 (68.2)	893.1 (23.3)	0.000
Muslim	427.9 (52)	395.2 (48)	823.1 (21.5)	0.688
Traditional	6.2 (57.9)	4.5 (42.1)	10.7 (0.3)	0.704
Others	5 (35.2)	9.2 (64.8)	14.2 (0.4)	0.926
Heard about FP at community				
Yes	851.9 (49.6)	865.9 (50.4)	1717.8 (44.8)	------
No	919.2 (43.4)	1198 (56.6)	2117.2 (55.2)	0.011
Media exposure				
Exposed	581.3 (47.7)	638.1 (52.3)	1219.4 (31.8)	------
Not exposed	1189.7 (45.5)	1425.9 (54.6)	2615.6 (68.2)	0.057

(FP)-family planning, (------) reference category

Majority (58%) of women: living in community with high contraceptive use rate, (61.4%) residing in Tigray, and (57.1%) residing in Adiss Ababa had high unscheduled contraceptive use discontinuation, whereas women living in low educated community and poor community had relatively low discontinuation rate of 45 and 44.7% respectively (see Table 3).

**Table 3:** Description and bivariate logistic analysis of community level factors of unscheduled contraceptive discontinuation, EDHS 2016 December 2018

Community Level Variables	Unscheduled Contraceptive Discontinuation			
	Yes (%)	No (%)	Total (%)	p-value
Region				
Tigray	189.9 (61.4)	119.3 (39.6)	309.2 (8.1)	0.000
Afar	3 (29.4)	7.2 (70.6)	10.2 (0.3)	0.002
Amhara	627.6 (50.7)	609.3 (49.3)	1236.9 (32.3)	0.000
Oromia	524.4 (44.8)	647.4 (55.2)	1171.8 (30.6)	0.076
Somali	1.8 (43.9)	2.3 (56.1)	4.1 (0.1)	0.894
Benshangul	15.8 (44.4)	19.8 (55.6)	35.6 (0.9)	0.120
SNNPR	256.7 (32.3)	538.8 (67.7)	795.5 (20.7)	0.000
Gambella	4.9 (43.7)	6.3 (56.3)	11.2 (0.3)	0.039
Harari	3.1 (43.7)	3.95 (56.3)	7.1 (0.2)	0.160
Adiss abab	134.2 (57.1)	100.7 (42.9)	234.9 (6.1)	0.081
Dire dawa	9.5 (51.9)	8.8 (48.1)	18.3 (0.5)	------
Type of residence				
Urban	448.8 (49.4)	458.9 (50.6)	907.7 (23.7)	
Rural	1322.2 (34.5)	1605.1 (65.5)	2927.3 (76.3)	0.003
Community contraceptive				
Low	467.2 (58)	337.8 (42)	805 (30)	0.000
High	1303.8 (43)	1726.2 (57)	3030 ()	------
Community education				
Low	1016.8 (45)	1242.4 (55)	2259.2 (58.9)	0.021
High	754.3 (47.9)	821.5 (52.3)	1575.8 (41.1)	------
Community media				
Not exposed	903.8 (44.1)	1145.5 (55.9)	2049.3 (53.4)	0.000
Exposed	867.2 (48.6)	918.5 (51.4)	1785.7 (46.6)	------
Community wealth				
Poor	838.5 (44.7)	1036.7 (55.3)	1875.3 (48.9)	0.000
Rich	932.5 (47.6)	1027.2 (52.4)	1959.7 (41.1)	------

*FV* health facility visit, *FW* field worker, ------ reference category

NB: any descriptive table is weighted, unless indicated as un-weighted

### Reasons of contraceptive discontinuation

Most (71.37%) of the discontinued methods were Injectables followed by long acting (13.21%) and pills (10.95%). Method related reasons were found to be the principal ones for unscheduled discontinuation of both short acting and long acting contraceptives. Of the reasons to discontinue a contraceptive utilization, 54.55% were method related (side effects, inconvenience, and needing better method). Contraceptive failure was reported highest in women of pill users (12%) followed by injectable users (3.3%) compared to the long acting methods. Only 6.45% women had a history of last method switched in to another method, while the unscheduled discontinuation is 46.18% (refer Table 4).

**Table 4:** Frequency distribution of reasons of discontinuation for different contraceptive methods, from-EDHS 2016, January 2018

Discontinued methods	Reason to discontinue a contraceptive						
	became pregnant (failure)	method related	Access	husband opposed	Unreliable reasons	other reasons	Total
Pill	23	106	13	1	39	12	194
Injectables	41	710	116	27	241	129	1264
Long acting	4	158	1	4	36	31	234
Male condom	0	4	0	0	16	4	24
Other modern	5	10	0	0	17	5	37
Traditional	3	8	0	0	7	0	18
Total	76	996	130	32	356	181	1771

Other reasons = (fatalistic, don’t know), method related reasons = (side effect, inconvenience, and needing better method)

### Multivariable multilevel logistic regression analysis

#### Measures of components of variance and heterogeneity

In the model building, the empty model (model-0), that had no predictor variable, showed that the proportion of variation on the unscheduled contraceptive discontinuation explained by the clustering effect was 11.12% using the logit-link and 13.5% using the probit-link; indicated that the presence of statistically significant variability on unscheduled contraceptive use discontinuation across the communities. The median odds ratio (MOR = 1.844) reflected that the odds of unscheduled contraceptive use discontinuation was 1.844 times higher in the persons with the higher propensity to the outcome of interest compared to those persons with lower propensity.

The reported likelihood-ratio test showed that there was highly statistically significant variability (*p*-value < 0.001) on unscheduled contraceptive discontinuation between women from different communities to favor a multilevel logistic regression over a standard logistic regression.

The estimated intercept of empty model was − 0.088756, and therefore; at an average community (whose specific intercept is equal to the fixed intercept), the probability of one episode unscheduled contraceptive use discontinuation within five years was 0.478.

In model-1, the MOR indicated that, 66.9% (MOR = 1.669) of increase in the odds of unscheduled contraceptive discontinuation among the women with higher propensity to the outcome was accounted by differences across communities. In addition the PCV in model-1 implied that less than one-third (29.68%) of the variation due to differences across communities (general contextual effect) on unscheduled contraceptive discontinuation was explained by individual level predictors, and the ICC and MOR values decreased to 8.1% and 1.671 respectively from the null-model.

The MOR (1.457) in model-2 revealed that the clustering effect accounts for 45.7% increase on the odds of unscheduled contraceptive discontinuation and in the final model (MOR =1.419) 41.9% increase in the odds of the outcome variable was attributed by differences across communities. The PCV on model-2 and model-3 revealed that 62.20 and 67.21% of the community variance in null-model was explained by adding community-level variables in model-2, and by adding both individual and community level variables in model-3 respectively (refer Table 5).

**Table 5:** measures of components of variance and heterogeneity on unscheduled contraceptive discontinuation, multilevel logistic regression analysis EDHS 2016, January 2018

RANDOM EFFECTS	MODEL-0	MODEL-1	MODEL-2	MODEL-3
Measure of general Contextual effect				
ICC	11.12%	8.08%	4.52%	3.94%
MOR	1.844	1.671	1.457	1.419
Var	0.4114	0.2893	0.1555	0.1349
PCV		29.68%	62.20%	67.21%
AUC % (95%CI)	75.35 (0.73835, 0.76859)	72.91 (0.71338, 0.74448)	69.89 (0.68255, 0.71530)	70.29 (0.68659, 0.71917)
MODEL Selection				
AIC	5227.386	5189.581	5118.615	5106.208
AUC% (Fixed effect only)	Reference	59.94	63.13	64.83
Log-likelihood (LR-test p-value)	− 2611.6929 (0.0000)	− 2567.79 (0.0000)	− 2541.3073 (0.0000)	− 2510.104 (0.0002)

*ICC* intra-class correlation, *MOR* median odds ratio, *Var* random intercept variance, *PCV* proportional change in variance, *AUC* area under the curve, *AIC* akaki’s information criteria, *CI* confidence interval

The LR-test stated that the multilevel logistic regression was significantly favored over a standard (ordinary) logistic regression for this clustered nature of data.

#### ROC curve as measure of general contextual effect and goodness of fit

When prediction is based on both fixed effects and posterior means of random effects, using predict probability, mu command in STATA, the AUC is used as a measure of general contextual effect and, the ICC and AUC are usually consistent (both, either high or low) [28]. In this study AUC, as a measure of general contextual effect, was found to be near consistent with ICC size which makes sense as expected.

**Fig. 3:**
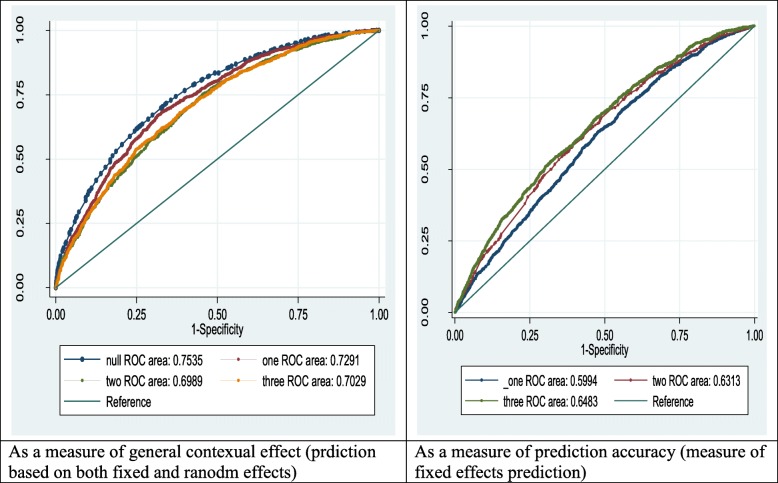
ROC curves as a measure of general contextual effect and goodness of fit, EDHS 2016, January 2018

Using predict probability, xb command in STATA, prediction is done based on only fixed effects. In this situation the AUC is a measure of goodness of fit which uses only fixed effect for prediction as AIC do. So, both are expected to give consistent decision. In this study, the final model was found to have relatively good fit, from both AIC and AUC values (refer Fig. 3).

#### Multilevel factors associated with unscheduled discontinuation

Household head, women’s occupation, religion and number of children per woman were statistically significant predictors associated with unscheduled contraceptive discontinuation in Model-1. Residing in low educated community, rich community, and community with low contraceptive rate and regions were found to be significant predictors of unscheduled contraceptive discontinuation in Model-2. This is just to illustrate the variables that were significant in model-1 and model-2, to see how those variables are modified to each other in the final best model (see Table 6).

**Table 6:** Multivariable multilevel logistic regression analysis of individual and community level factors associated with unscheduled contraceptive discontinuation, EDHS 2016, January 2018

Fixed Effect Of Variables	AOR (95% CI)		
	MODEL-1	MODEL-2	MODEL-3
Age group			
15–24	0.885(0.72, 1.088)		1.083(0.880, 1.333)
25–34	1		1
35–49	0.891(0.737, 1.077)		0.933(0.712, 1.222)
Media exposure			
Not exposed	0.939(0.792, 1.116)		0.941(0.788, 1.123)
exposed	1		1
Heard about FP at Community event			
Not heard	0.881(0.762, 1.018)		0.910(0.787, 1.052)
heard	1		1
Number of children			
< =1	0.718 (0.592, 0.872)		0.682 (0.561, 0.828)*
> =2	1		1
Household head			
Male	1		1
Female	1.316 (1.109, 1.561)		1.281 (1.079, 1.520)*
Respondent’s occupation			
No occupation	0.793(0.658, 0.955)		0.812(0.673, 0.979)*
Professionals	1		1
Services	1.016(0.727, 1.419)		1.017(0.727, 1.421)
Skilled manual	0.812(0.659, 0.999)		0.834(0.676, 1.029)
Unskilled manual	0.809(0.479, 1.369)		0.739(0.439, 1.241)
Others	0.653(0.424, 1.006)		0.641(0.0.416, 0.987)
Household wealth index			
Poorest	0.834(0.631, 1.103)		0.753,(0.567, 0.997)*
Poorer	0.917(0.710, 1.183)		1.206(0.912, 1.594)
Middle	1		1
Richer	0.984(0.768, 1.259)		1.269(0.946, 1.702)
Richest	1.064(0.830, 1.364)		1.089(0.758, 1.566)
RELIGION			
Orthodox	1		1
Catholic	0.723(0.299, 1.747)		1.176(0.489, 2.828)
Protestant	0.595 (0.481, 0.734)		0.822(0.646, 1.046)
Muslim	0.985(0.813, 1.193)		1.129(0.921, 1.383)
Traditional	1.339(0.263, 6.832)		1.324(0.260, 6.745)
Others	1.053(0.303, 3.654)		1.207(0.343, 4.242)
Region			
Tigray		1.847 (1.264, 2.701)	1.883 (1.267, 2.798)*
Afar		0.379 (0.202, 0.713)	0.370 (0.196, 0.697)*
Amhara		1.394(0.956, 2.031)	1.333(0.906, 1.963)
Oromia		0.901(0.610, 1.329)	0.903(0.611, 1.963)
Somali		0.718(0.270, 1.911)	0.655(0.245, 1.754)
Benshangul		0.991(0.653, 1.505)	0.924(0.605, 1.412)
SNNPR		0.691 (0.419, 0.914)	0.682(0.451, 1.031)
Gambella		0.887(0.581, 1.355)	1.004(0.653, 1.544)
Harari		0.804(0.523, 1.234)	0.768(0.499, 1.182)
Adiss abab		1.398(0.979, 1.997)	1.474(1.030, 2.108)*
Dire dawa		1	1
Community contraceptive			
Low		1.984 (1.651, 2.383)	1.945 (1.618, 2.339)*
High		1	1
Community education			
Low		1.313 (1.052, 1.639)	1.246(0.998, 1.556)
High		1	1
Community wealth			
Poor		0.748 (0.595, 0.942)	0.763 (0.596, 0.977)*
Rich		1	1
Community media			
Not exposed		0.919(0.735, 1.151)	0.957(0.763, 1.201)
exposed		1	1
Residence			
Rural		0.835(0.636, 1.095)	0.768(0.557, 1.06)
Urban		1	1

(1)-reference category, (*) – indicates significant predictors

#### Final best model (Model-3): cross adjusted association

In final model (model-3), both the individual level and community level variables were added at the same time; in which community-level factors modify the association between individual level factors and unscheduled contraceptive use discontinuation.

After adjusting the individual and community level characteristics and after fixing the random effects, women who were a head of household were 1.28 (AOR = 1.275, 95%CI 1.075–1.513) more likely to experience unscheduled contraceptive discontinuation compared to women of male headed households.

Keeping other variables constant, women who had no work were 19.4% (AOR = 0.806, 95%CI 0.669, 0.973) lower to have unscheduled contraceptive discontinuation when compared to professional women (government and NGOs employee). The odds of unscheduled contraceptive use discontinuation was 24.7% (AOR = 0.753, 95%CI 0.567, 0.997) lower on women living in poorest house hold income quintile compared to middle house hold income quintile.

After holding the other individual and community level variable constant, residing in Tigray region was 1.939 times (AOR = 1.939, 95%CI 1.306, 2.879) higher, residing in Afar region was 62.5% (AOR = 0.375, 95%CI 0.199, 0.707) lower and residing in Adiss-Ababa administrative city was 1.507 times (AOR = 1.507, 95%CI 1.054, 2.154) higher to discontinue contraceptive use despite the desire to avoid pregnancy compared to Dire-dawa residents. Comparing women residing in community with low contraceptive utilization rate to women living in community with high contraceptive utilization rate, the odds of unscheduled contraceptive discontinuation was about 2 times higher (AOR = 1.922, 95%CI 1.599, 2.309) among the former ones. Adjusting for other variables, the odds of unscheduled contraceptive discontinuation was 22.6% (AOR = 0.774, 95%CI 0.605–0.990) less likely for women residing in poor community compared to women residing in rich community. Controlling for other variables, women who had <=1 child were 31.8% (AOR 0.682, 95%CI 0.561, 0.828) lower to have unscheduled contraceptive use discontinuation compared to women who had two and above children (refer Table 6).

Those multilevel factors were tested for multi-collinearity and no variable was found to have problematic multi-collinearity with VIF value > 10.

#### Summary measures of effect for cluster-level covariates

The interval odds ratios (IOR) of the three significant community level predictors (community wealth, community contraceptive use rate and region) were 0.3925–1.486, 0.999–3.786, and 0.968–3.664 respectively. Here the IOR provided complementary information to the information provided by usual AOR in comparison to the residual community level variation, those significant variables were not important to understand community level variation in the individual propensity for experiencing unscheduled contraceptive discontinuation i.e. the effect of the those higher-level variables is not strong given the residual between-community variation.

This analysis of IOR indicates that there are strong community-level effects on contraceptive use discontinuation which were not accounted for by the community-level variables included in the model. Further investigation of these community-level effects is an important area of research.

## Discussion

This study delivers important insights in to the unscheduled contraceptive discontinuation from Ethiopian demographic and health survey of 2016 data. The objective of this study was assessment of reasons and multilevel factors associated with unscheduled contraceptive use discontinuation. The discussion would be focusing on methods discontinued, reasons of discontinuation, individual level factors and community level factors.

NB: The discussion about multilevel factors is all about the findings from the final model (the best model selected using both AUC and AIC).

### Methods discontinued

IUD and implant discontinuation rates were lowest compared to others. This is like finding of the study conducted in Maryland, low income countries, urban Senegal and 60 DHS of countries [1, 4, 29, 30]. This lowest discontinuation of long acting reversible contraceptive could be due to low side effects and greater efficacy compared to the hormonal contraceptives, and the need of health care provider for removal might give another chance to get further counseling from health care professionals.

### Reasons for discontinuation

In this study, among women who have unscheduled discontinuation, more than half of discontinuations were for the reason of failure or method-related problems which is similar to the study done in low income countries [29], and the principal reason for unscheduled contraceptive discontinuation was method related reasons and this is in agreement with the studies done in London, India-2016, Senegal, Cambodia, and Pakistan [1, 30–33]. This might be because of increasing women’s interests to concern more about their health and therefore; go to search for other better methods, being stressed with minor side effects and needing more convenient contraceptive method.

This study implied that discontinuation due to access was few which is similar to the studies done in 60 DHS of countries, Cambodia, Bangladesh, Guatemala, and Calverton [4, 32, 34–36]. Family planning is coordinated by different NGOs and governmental organizations freely, and this could improve the access. Other reasons are found to be the main reasons of condom use discontinuation compared to method related reasons which is similar to the study done in India [33].

### Significant individual level factors

In this study, women of reproductive age who had more children were found to be more likely to have unscheduled contraceptive discontinuation. This is in agreement with study done in India and in Bangladesh [33, 36], and this could be similar to the critical review study done in 2015 that revealed women aged 25–34 years old were more likely to have discontinued than women aged less than 20 years [37] who could have lower number of children. This is may be due to the reason of thinking as reaching menopausal period by the older mothers. A study done in Bangladesh stated that contraceptive failure and abandonment while in need are more likely among middle age women compared to the younger counter parts and among women with more children [38]. In addition the older mothers may come across different contraceptive method that may be faced different method related problems, and this in turn may make them to be non-tolerant to minor inconveniences in the later methods.

This study implied that the odds of unscheduled contraceptive discontinuation were higher in professionals than those who had no occupation; this could be parallel to the study done in Bangladesh and critical review study of 2015 which implied that discontinuation was higher among educated women compared to women with no formal or with less education [36, 37]. Myths and rumors are often created and reinforced by women’s social interactions at places where they gather and exchange information [39]. So those professional women may have higher social interactions to get myths and misconceptions to discontinue their utilization. In addition, Different researches showed that the highest knowledge, but low contraceptives practice; making the situation a serious challenge in which knowledge on contraceptives did not match with high contraceptive practice [40–42]. This indicates that not access and awareness only are enough to increase contraceptive practice, but improving counseling about contraceptive side effects; generally about method related problems and switching are necessarily important. Thus, the reason to professional women to have higher discontinuation might be poor switching encouragement, and poor counseling to address method related problems. More over; the professionals may also easily be dissatisfied by the family planning services to discontinue their contraceptive use, as their expectation may be beyond the actual quality when compared to the non-professional.

The odds of unscheduled contraceptive discontinuation was higher in these living in middle income quintile than living in poorest income quintile in line to the study done in Bangladesh and Pakistan [31, 38]. Most of reproductive age women, who have more interpersonal interaction, know incorrect information about family planning methods; and these women who have heard false and misleading information would have a negative attitude about family planning practicing at the time they heard [43, 44]. Thus the women with middle income may have greater chance of interpersonal interactions to hear this false and misleading information, than the poorest women, to discontinue their contraceptive use.

This study also stated that women heading a household were found to have higher odds of unscheduled discontinuation compared to their counter parts (women who are not heading a household). This heading of household may increase the participation of women in social issues and may increase their chance to get contraceptive related information that could be accurate, misconceptions or rumors; and may also increase their concern to their health. Hence, those women may be sensitive to minor method inconveniences which may make them to have high discontinuation compared to women having a life of individualism. In addition, the women who are heading a house hold may be those who are divorced, not married and whose husbands are away or died. Such women are prone to unreliable reasons of discontinuation (like infrequent sex/husband away, marital dissolution/separation).

### Significant community level factors

This study showed, women residing in community with higher contraceptive use rate had lower odds of unscheduled contraceptive use discontinuation compared to women living in community with lower contraceptive use rate which is similar to studies done in Calverton-Maryland, sixty DHS countries and low income countries [4, 29, 35]. This might be because of women living in communities with greater contraceptive rate may receive more peer support to continue contraceptive use if they have problems, and may receive more peer pressure not to have unwanted pregnancies. In addition, these communities may positively relax their norms and cultures towards continuous utilization of contraceptive use, as users increase.

Region, similar to several studies (33, 38, 41, and 46), was found to be significant predictors of unscheduled contraceptive use discontinuation. This is may be due to different commitments towards reducing discontinuation among the different regions; and in addition to that, it could be due to the difference in knowledge and socioeconomic characteristics among the different regions.

Community wealth, similar to studies [34, 42], was found to be significant predictors of unscheduled contraceptive use discontinuation from the AOR. The wealthier communities may have higher knowledge on family planning, but different researches showed that the highest knowledge on contraceptives did not match with high contraceptive practice [40–42]. In addition, the wealthier communities may have more social interactions in which myths and rumors, which lead to discontinuation, could be exchanged.

## Strength and limitation

### Strength

- DHS data has individual (women) level sampling weight that is used to weight the descriptive part to make it more representative.

### Limitation

- DHS data has no cluster level weighting which is, in multilevel modeling, necessary for performing weighted analysis (for the analytical part).
- Some variables included in the model might not be collected at the same date of the event occurrence

## Conclusion

The prevalence of unscheduled contraceptive use discontinuation was 46.18%. Of the last contraceptive methods discontinued while in need, majority were Injectables followed by long acting and pills. The principal reason of contraceptive use discontinuation was method related problems. Unscheduled contraceptive use discontinuation was higher among women residing in Tigray and Addis Ababa (compared to Dire-dawa), higher among women living in community with low contraceptive use rate (compared to high), higher among women having many children (compared to having 1-child), lower among women with poorest income quintile (compared to middle), lower among women not heading household (compared to women heading household), and lower among women with no occupation (compared to professionals). IOR-80% implied the presence of other important community level variables, not considered in the model, that need to be considered. The outcome was common in groups who could have more social interactions and knowledge on which myths and rumors are common. So strengthening the efforts to reduce contraceptive use discontinuation and quality of contraceptive service provision could be important.

## Supplementary information

**Additional file 1.** STATA output for random slope of the variable women’s education

## Data Availability

The data set used in this study had not been released to public. According to relevant regulations, the data cannot be shared publicly. However, the extracted data can be obtained from the corresponding author up on reasonable request. The source data can also be obtained from major DHS through Email if there is satisfactory reason to access the data. But this data is not publicly available.

## Abbreviations

AIC: Akaiki’s Information Criteria
AUC: Area under the Receiver Operating Characteristic
BIC: Bayesian Information Criteria
CPR: Contraceptive Prevalence Rate
CSA: Central Statistical Agency
DHS: Demographic Health Survey
EDHS: Ethiopian Demographic Health Survey
ICC: Intra-Cluster Correlation Coefficient
IOR: Interval Odds Ratio
IRB: Institutional Review Board
IUDs: Intrauterine Devices
LARC: Long Acting Reversible Contraceptives
MLRM: Multilevel Logistic Regression Model
MOR: Median Odds Ratio
PCV: Proportional Change in Variance
ROC: Receiver Operating Characteristic
SDG: Sustainable Development Goal,
VIF: Variance Inflation Factor
VPC: Variance Partition Coefficient
WHO: World Health Organization
