# Supplementary material for: Reasons and multilevel factors associated with unscheduled contraceptive use discontinuation in Ethiopia: evidence from Ethiopian demographic and health survey 2016
Source: BMC Public Health. 2019 Dec 27;19:1745. doi: 10.1186/s12889-019-8088-z (PMC6935182; doi:10.1186/s12889-019-8088-z)
Supplement: Supplementary file 1 — Additional file 1. STATA output for random slope of the variable women’s education [file 12889_2019_8088_MOESM1_ESM.docx]

- 1. Additional file

STATA output for random slope of the variable women’s education
